# Expanding the Phenotype of the *FAM149B1*-Related Ciliopathy and Identification of Three Neurogenetic Disorders in a Single Family

**DOI:** 10.3390/genes12111648

**Published:** 2021-10-20

**Authors:** Sandy Siegert, Gabriel T. Mindler, Christof Brücke, Andreas Kranzl, Janina Patsch, Markus Ritter, Andreas R. Janecke, Julia Vodopiutz

**Affiliations:** 1 Department of Pediatrics and Adolescent Medicine, Division of Pediatric Pulmonology, Allergology and Endocrinology, Comprehensive Center for Pediatrics, Medical University of Vienna, 1090 Vienna, Austria; sandy.siegert@meduniwien.ac.at; 2 Department of Pediatric Orthopaedics, Orthopaedic Hospital Speising, 1130 Vienna, Austria; Gabriel.Mindler@oss.at (G.T.M.); Andreas.Kranzl@oss.at (A.K.); 3 Vienna Bone and Growth Center, 1090 Vienna, Austria; janina.patsch@meduniwien.ac.at; 4 Department of Neurology, Medical University of Vienna, 1090 Vienna, Austria; christof.bruecke@meduniwien.ac.at; 5 Laboratory for Gait and Movement Analysis, Orthopaedic Hospital Speising, 1130 Vienna, Austria; 6 Department of Biomedical Imaging and Image-Guided Therapy, Medical University of Vienna, 1090 Vienna, Austria; 7 Department of Ophthalmology, Medical University of Vienna, 1090 Vienna, Austria; markus.ritter@meduniwien.ac.at; 8 Department of Pediatrics I, Medical University of Innsbruck, 6020 Innsbruck, Austria; Andreas.Janecke@i-med.ac.at; 9 Division of Human Genetics, Medical University of Innsbruck, 6020 Innsbruck, Austria

**Keywords:** *KMT2B*, *POLG2*, *FAM149B1*, ciliopathy, duane syndrome, precision medicine, deep brain stimulation, multiple genetic disorders, olfactory bulb aplasia, Joubert syndrome

## Abstract

Biallelic truncating *FAM149B1* variants result in cilia dysfunction and have been reported in four infants with Joubert syndrome and orofaciodigital syndrome type VI, respectively. We report here on three adult siblings, 18 to 40 years of age, homozygous for the known *FAM149B1* c.354_357delinsCACTC (p.Gln118Hisfs*20) variant. Detailed clinical examinations were performed including ocular and gait analyses, skeletal- and neuroimaging. All three patients presented with neurological and oculomotor symptoms since birth and mild skeletal dysplasia in infancy resulting in characteristic gait abnormalities. We document mild skeletal dysplasia, abnormal gait with increased hip rotation and increased external foot rotation, ataxia, variable polydactyly, ocular Duane syndrome, progressive ophthalmoplegia, nystagmus, situs inversus of the retinal vessels, olfactory bulb aplasia, and corpus callosal dysgenesis as novel features in *FAM149B1*-ciliopathy. We show that intellectual disability is mild to moderate and retinal, renal and liver function is normal in these affected adults. Our study thus expands the *FAM149B1*-related Joubert syndrome to a mainly neurological and skeletal ciliopathy phenotype with predominant oculomotor dysfunction but otherwise stable outcome in adults. Diagnosis of *FAM149B1*-related disorder was impeded by segregation of multiple neurogenetic disorders in the same family, highlighting the importance of extended clinical and genetic studies in families with complex phenotypes.

## 1. Introduction

Ciliopathies comprise a genetically and clinically heterogenous group of disorders caused by both motile and non-motile cilia dysfunction. While motile-cilia dysfunction results in infertility, chronic respiratory symptoms and lateralization defects, non-motile cilia dysfunction may result in a wider phenotypic spectrum including congenital anomalies (brain/heart/skeletal system/midline defects), progressive organ dysfunction (retina/liver/kidney/ear), and organ laterality defects. For a review, see references [1,2].

Joubert syndrome (JS) is one of the well-known non-motile ciliopathies and is defined by characteristic midbrain-hindbrain malformations, giving the midbrain an appearance of a molar tooth in an axial MRI-section: the molar tooth sign (MTS) [3,4]. Oculomotor disturbances, abnormal respiratory pattern, muscular hypotonia, ataxia, and developmental delay are characteristic features in JS [3,5]. These neurological features can be complicated by a large spectrum of ciliopathy-associated organ defects such as retinopathy, progressive renal and liver failure, or skeletal disorder [6]. Orofaciodigital syndrome type VI (OFD VI) partially overlaps with JS and is defined by the triad of MTS, orofacial and digital features such as cleft lip and palate, intraoral frenula, mesoaxial polydactyly, brachy- and clinodactyly [7,8].

Recently biallelic loss-of-function variants in the family with sequence similarity 149 member B1 gene (*FAM149B1*) have been identified in four children causing cilia dysfunction and resulting in a phenotypic overlap of JS and OFD VI [9]. Developmental delay, intellectual disability, oculomotor apraxia, ptosis, and mesoaxial polydactyly were constant features in the four previously reported *FAM149B1*-related ciliopathy patients, while MTS and orofacial features were variably present [9]. Due to the small number of reported patients and their young age at the time of publication, the phenotypic spectrum and the adult phenotype of the *FAM149B1*-related ciliopathy remained unclear.

Here we report the detailed clinical findings of three adults who are homozygous for the originally reported *FAM149B1* p.Gln118Hisfs*20 variant and thereby expand the neurological, ocular and skeletal phenotype of the *FAM149B1*-reated ciliopathy beyond childhood. Diagnosis of *FAM149B1*-related disorder was impeded by segregation of two additional neurogenetic disorders in the same family, highlighting the importance of extended clinical and genetic studies in families with complex phenotypes to enable individual and disease-specific management and counseling.

## 2. Materials and Methods

Clinical studies are described in Supporting Information. Genomic DNA was extracted from peripheral blood leukocytes from all participants by standard procedures. Whole exome, paired-end sequencing (WES) was performed after target enrichment in 1 µg of genomic DNA with the Roche-Nimblegen’s SeqCap EZ Exome v2 kit in P3 as described [10] and with the Agilent SureSelectHuman All Exon 61 Mb Capture kit (Agilent Technologies, Santa Clara, CA, USA) in P5 [11] using an Illumina Hi-Seq2000 and Illumina Hi-Seq4000 platform, respectively. Variants identified through WES were filtered for autosomal recessive (homozygous and compound heterozygous variants) and autosomal dominant mode of inheritance, predicted effect on protein expression (missense, nonsense, splice site, in-frame indels, and frameshift), and allele frequency of <0.005 in the dbSNP (http://www.ncbi.nlm.nih.gov/projects/SNP/, accessed on 5 March 2020), National Heart, Lung, and Blood Institute (http://evs.gs.washington.edu/EVS, accessed on 5 March 2020), and Exome Aggregation Consortium (http://exac.broadinstitute.org/, accessed on 5 March 2020) databases. Missense variants were evaluated in silico for pathogenicity by PolyPhen-2 (http://genetics.bwh.harvard.edu/pph2, accessed on 5 March 2020) and CADD (http://cadd.gs.washington.edu/score, accessed on 5th March 2020). Sanger sequencing of mutated *FAM149B1*, *POLG2,* and *KMT2B* exons was applied in all 12 siblings and in the mother of one large consanguineous family for segregation analysis of variants. Variants designations are based on the NCBI transcript reference sequences NM_173348.2 for *FAM149B1*, NM_007215.3 for *POLG2*, and NM_014727.3 for *KMT2B*, respectively.

## 3. Results

### 3.1. Clinical Characteristics

**Figure 1:**
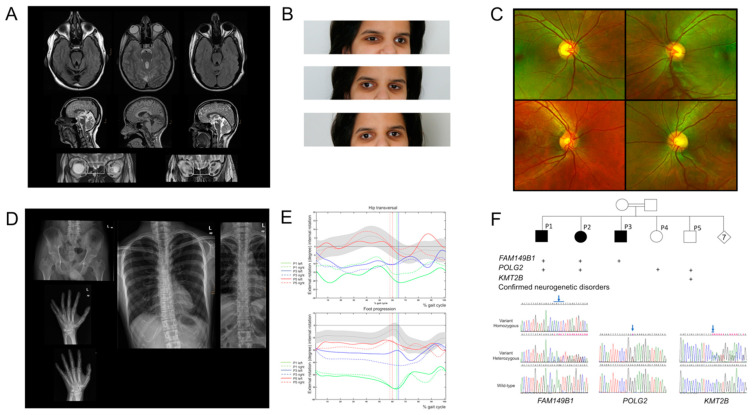
Clinical features in three siblings with biallelic *FAM149B1* variants. (**A**): Brain MRI in P1, P2, P3 (left, middle, right column) with MTS (upper row), cerebellar hypoplasia of the upper vermis and callosal dysgenesis (middle row). Olfactory imaging (bottom row) with olfactory bulb aplasia in P1 (left box) versus normal olfactory bulb in P3 (right box). (**B**): Bilateral Duane syndrome during childhood with compensatory head tilt in P2: limited abduction of left eye with induced ptosis on adduction of right eye (upper panel) and limited abduction of the right eye with induced ptosis of left eye (middle panel). Normal palpebral fissures of both eyes in primary position (bottom panel). (**C**): Fundus photographs with situs inversus of retinal vessels in P2 (upper row) and situs solitus in a healthy control (lower row) for comparison. (**D**): Radiographic features of mild skeletal dysplasia and variable digital features. (**E**): Gait analysis with increased hip rotation by relative motion of the femur to the pelvis (upper panel) and increased external foot rotation by absolute motion of the foot to the walking direction (lower panel) in the transversal plane in P1, P3, and P5. Gray bands indicate the reference group (mean + 1 SD), Color coded vertical line splits the gait cycle into stand and swing phase. (**F**): Simplified pedigree with ciliopathy-affected individuals indicated by full symbols, and segregation analysis and sequence chromatograms for *FAM149B1*, *POLG2*, and *KMT2B* variants detected in the same family. “+”, “present”.

Three siblings (P1, P2, and P3), 18 to 40 years of age, from a large consanguineous family from Saudi Arabia were referred to evaluate a syndromic form of developmental delay, finally diagnosed as *FAM149B1*-related JS. Brain MRI demonstrated the classic MTS and hypoplasia of the upper cerebellar vermis in all three patients, mild to moderate dysgenesis of the corpus callosum in two patients (shortening and thickening with absent isthmus in P1 and minor structural abnormalities in P3), and olfactory bulb aplasia in P1 and P2 (Figure 1A). These patients presented with muscular hypotonia, delay in their developmental milestones, mild cerebellar ataxia, mild to boundary moderate intellectual disability with limited expressive language skills, and mild need for assistance in all activities of daily living. All patients completed a school for children with special needs and P1 was able to speak and write simple sentences in Arab and German but had difficulties to calculate beyond the number range over 20. 

Oculomotor disturbances were noted in all three patients since birth and Duane syndrome and nystagmus were diagnosed in infancy. These oculomotor disturbances were masked in all three patients in late childhood by progressive ophthalmoplegia, resulting in severe, predominantly horizontal ophthalmoplegia in late adolescence in all three patients and in complete ophthalmoplegia in the fourth decade in the oldest P1 (Figure 1B). Situs inversus of the retinal vessels (Figure 1C), ptosis, and severe myopia were diagnosed in one patient each, while detailed ocular investigation by fundus photographs, optical coherence tomography, electroretinogram, and visual evoked potential revealed otherwise normal results at 18, 24, and 40 years of age (Table 1 and Table 2). 

All three patients presented with a mild skeletal dysplasia with trident acetabular roofs and protrusio acetabuli, resulting in characteristic gait abnormalities in infancy with increased hip rotation and external foot rotation in clinical gait analysis (Figure 1D). Computer based gait analysis in P1 and P3 showed a significantly reduced Gait Deviation Index (Table 2), and proved increased hip rotation and external foot rotation (Figure 1E). Mild clino- or brachydactyly V was noted bilaterally in two patients, mesoaxial polydactyly with Y-shaped metacarpalia unilaterally in one patient, and premature and overt calcification of costal cartilage in two patients (Figure 1D). 

Neither subtle clefts of the midline, renal or hepatic involvement, obesity, nor hypogonadism were present in any of these patients at age 18, 24, and 40 years and there was no history of breathing abnormalities or seizures (Figure 1, Table 1 and Table 2).

### 3.2. Identification of a Homozygous Truncating JS-Causing FAM149B1 Variant

WES in P3 and familial segregation analysis identified homozygosity for the reported truncating *FAM149B1* variant c.354_357delinsCACTC (p.Gln118Hisfs*20) in all affected patients P1, P2, and P3 as the only deleterious variant causing a monogenic disease and segregating with this disease phenotype in the family (Figure 1F, Supplementary Tables S1 and S2). This *FAM149B1* variant was originally delineated as c.356_357delAG (p.Lys119Ilefs*18) [9].

### 3.3. Identification of Two Additional Neurogenetic Disorders in the Same Family

During diagnostic work up for syndromic developmental delay in this large consanguineous pedigree, two further neurogenetic diseases-segregating independently of each other and of the *FAM149B1*-related phenotype in the family were detected by clinical investigations and supported by genetic testing (Figure 1F). 

Mild myopathy, exercise intolerance, exercise induced lactate elevation, and intermitted juvenile onset strabismus pointed to a mitochondrial disorder in four siblings P1, P2, P4, and P5. Autosomal-dominant *POLG2*-related mitochondrial disorder (progressive external ophthalmoplegia with mitochondrial DNA deletions, autosomal dominant 4; PEOA4) was indicated as a diagnosis by identifying heterozygosity for the novel truncating *POLG2* variant c.886G>T (p.Gly296*) by WES in P5 and by its segregation with the mitochondriopathy-phenotype in this family. P5 additionally presented progressive, juvenile onset dystonia with predominant cranio-cervical involvement and was finally diagnosed with *KMT2B*-related childhood onset dystonia (Dystonia 28; DYT28) by identification of the novel de-novo c.5697del (p.Thr1900Glnfs*34) *KMT2B* variant. (Figure 1F) Clinical features of affected siblings and disease-specific diagnostic clues and pitfalls for *FAM149B1, POLG2,* and *KMT2B-* related neurogenetic disorders are shown in Table 2.

## 4. Discussion

We report three adult siblings with homozygosity for the known *FAM149B1* p.Gln118Hisfs*20 variant and compare their clinical features to the four previously reported pediatric patients with an overlapping phenotype of JS and OFD VI, of whom three Saudi Arabian patients were homozygous for the same truncating *FAM149B1* variant [9]. All seven patients presented with developmental delay, muscular hypotonia, oculomotor apraxia, and strabismus, whereas the MTS was present in five of six patients in whom brain MRI was obtained [9]. Unlike to the previously reported patients [9], ptosis and polydactyly were present in one patient each in the study presented here and OFD VI-related oral anomalies were not present, indicating phenotypic variability in *FAM149B1*-related JS. This is in accordance with intra- and inter-familial phenotypic variability in other subtypes of JS [3,5,6,12].

Cerebellar ataxia, olfactory bulb aplasia and corpus callosal dysgenesis are novel neurological features associated with *FAM149B1*-related JS and all but olfactory aplasia are common features in other subtypes of JS [3,5,9]. Support for olfactory aplasia due to *FAM149B1* dysfunction comes from mutations in other genes, associated with cilia function or structural integrity of cilia, and resulting in olfactory dysfunction, such as hypo- or anosmia in Bardet-Biedel-syndrome (BBS) [13,14,15], polycystic kidney disease [15], or rarely in OFD VI [8] and JS [16]. Olfactory dysfunction can easily be missed by clinical investigation and olfactory bulb volume evaluation is usually not applied in brain MRI, indicating that olfactory dysfunction and volume anomalies might be more common in ciliopathies than previously suggested, as it was shown in BBS. [14,16] While olfactory dysfunction is emerging as a characteristic ciliopathy feature, the detailed molecular mechanism of olfactory dysfunction in ciliopathies remains poorly understood [1,16] and putative molecular mechanism in *FAM149B1*-associated olfactory dysfunction have to be investigated in further studies.

While several brain-related oculomotor disturbances have been reported in JS [12,17], progressive ophthalmoplegia has not been reported so far, but is a constant feature in all three adult siblings reported here. WES in P3 did not reveal pathogenic variants in known genes for progressive external or brain-related ophthalmoplegia (Supplementary Tables S1 and S2), indicating *FAM149B1* to be causative for progressive ophthalmoplegia in these three siblings. Applying serial brain MRI and ocular investigation in adult JS patients might help to further characterize progressive ophthalmoplegia as a JS-related oculomotor disturbance. Situs inversus of the retinal vessels—a rare congenital abnormality characterized by emergence of the retinal vasculature in an anomalous, nasal direction, followed by a sharp turn towards the temporal direction [18]—was noted in one patient and might reflect situs defects in ciliopathies. Situs defects are common in motile ciliopathies but occurs also in non-motile ciliopathies such as Ellis-van Creveld or Jeune-syndrome, and have been reported in rare cases of JS and OFD [19]. Otherwise the ocular abnormalities in these three siblings are in accordance with the high frequency of ocular symptoms in JS [12,20] and importantly retinopathy was excluded in adulthood in all, indicating that *FAM149B1*-related ciliopathy is not associated with progressive retinal degeneration.

Mild skeletal dysplasia, resulting in characteristic gait abnormalities, is a novel feature in *FAM149B1*-related JS and was confirmed by skeletal radiographs in all three siblings displaying mild radiological hallmarks of skeletal ciliopathies such as trident acetabular roofs, protusion acetabuli, and variable digital features. Skeletal involvement is a common feature in ciliopathies and its severity ranges from lethal to very mild. [21,22,23]. The absence of thoracic hypoplasia and of short stature in these siblings supports mild skeletal involvement in *FAM149B1*-related ciliopathy, highlighting the importance of performing skeletal radiographs to diagnose skeletal involvement.

In summary, aggregate data from the previously reported patients [9] and from this study expands the clinical spectrum in seven patients with *FAM149B1*-related JS to a mainly neurological and skeletal ciliopathy phenotype with prominent oculomotor disturbances but otherwise stable outcome in adults. So far, only biallelic truncating *FAM149B1* variants have been reported, and identification of further biallelic variants will be needed to define the phenotypic spectrum in *FAM149B1*-related ciliopathy. Detection of the *FAM149B1* p.Gln118Hisfs*20 variant in four Saudi Arabian families suggests it is a founder variant.

Three different monogenic neurological disorders with predominant pediatric onset of movement disorder and oculomotor disturbances have been identified in this family, segregating independently of each other: autosomal recessive *FAM149B1*-related ciliopathy with neonatal onset of oculomotor disturbances and infantile onset of ataxia; autosomal dominant *POLG2*-related mitochondriopathy, which has been associated with progressive external ophthalmoplegia in the literature [24], but manifests mainly with exercise intolerance, mild myopathy, and intermittent strabismus in this family; and autosomal dominant *KMT2B*-related childhood onset dystonia. (Figure 1F, Table 2)

Accurate diagnosis of the phenomenon of multiple genetic disorders (MGD) in the same patient has been facilitated by massively parallel sequencing technologies and MGD is found in about 5% of patients in whom WES was informative [25]. In contrast to MGD in the same patient, MGD in the same family is still a diagnostic challenge because massively parallel sequencing technologies are usually not applied to all family members and because several family members can be affected by a different number of MGD. Therefore, applying massive parallel sequencing technologies in a subset of family members implies the risk of underdiagnosing MGD in the same family on a genetic level. Regarding the gaining importance of precision medicine in genetic diseases, it is urgent to identify all genetic causalities for complex phenotypes in families to enable individual and disease-specific treatment decisions and proper counseling for each family member [25]. Both additional genetic diagnoses in this consanguineous family are not autosomal recessive. This is in line with previous work [26] and emphasizes to consider all types of inheritance when performing genetic testing and upon counseling consanguineous families; this holds true whether or not one autosomal recessive disease was already identified in a given family [27].

Here, MGD for *FAM149B1*-, *POLG2*-, and *KMT2B*-related disorders in the same family was finally established by serial and detailed clinical examination of the whole family and in close collaboration of specialized clinicians and a human geneticist interpreting genetic and clinical family data (Table 2). Awareness for blended phenotypes and detailed clinical phenotyping of all family members to define affected and non-affected family members prior to genetic analysis are critical to decide in whom massively parallel sequencing methods should be applied. In this family, P3 and P5 were chosen for WES as the clinical phenotyping in P3 excluded mitochondrial disorder, but indicated the *FAM149B1*-related phenotype by neonatal onset of neurological and oculomotor symptoms. P5 was chosen to prove mitochondriopathy on a genetic level and because he was the only family member presenting with progressive childhood-onset dystonia. Genetic confirmation of both *POLG2*- and *KMT2B*-related neurological diseases in P5 enabled disease specific interventions such as globus pallidus internus deep brain stimulation and mitochondriopathy-related anesthetic consideration at age 21 years, resulting in significant improvement of motor function and disability, similar to previously reported efficacy of deep brain stimulation in *KMT2B*-related dystonia [28]. Beside genetic etiology, disease duration, body distribution, and the presence or absence of orthopedic deformities are factors that may predict the outcome of deep brain stimulation in monogenetic dystonias and should be included when counselling patients [29].

Other genetic disorders in this large family might have been missed as massively parallel sequencing technologies have been applied only in 2 out of 15 family members, but detailed clinical phenotyping of the whole family did not indicate other genetic disorders.

## 5. Conclusions

This study is the second report on *FAM149B1*-related ciliopathy and the first report on adult patients and expands the clinical spectrum and should increase awareness of this rare ciliopathy. We report mild skeletal dysplasia, olfactory bulb aplasia, corpus callosum dysgenesis, ataxia, ocular Duane syndrome, progressive ophthalmoplegia, nystagmus and situs inversus of the retinal vessels as novel clinical features in *FAM149B1*-related JS and exclude progressive renal, hepatic or retinal failure in these adults.

Identification of three neurogenetic disorders in the same family, presenting with overlapping phenotypes, highlights the importance of extended clinical and genetic studies in families with complex phenotypes to allow appropriate counseling and precision medicine for each family individual.

## Figures and Tables

**Table 1:** Ciliopathy features in seven patients with biallelic truncating *FAM149B1* variants.

Clinical Features	P1	P2	P3	Previously Reported	Total
Age	40 y	25 y	18 y	2 y, 3 y, 4 y, 17 y	2 y to 40 y
Sex	m	f	m	m = 4, f = 0	m = 6, f= 1
**Central Nervous System Features**					
MTS	+	+	+	2/3	5/6
Olfactory aplasia	+	+	−	0/3	2/6
Corpus callosal dysgenesis	+	−	+	0/3	2/6
DD/ID	+	+	+	4/4	7/7
OMA/strabismus	+	+	+	4/4	7/7
Ptosis	+	−	−	4/4	5/7
Muscular hypotonia	+	+	+	1/4	4/7
Ataxia	+	+	+	0/4	3/7
Duane Syndrome	+	+	+	0/4	3/7
Nystagmus	+	+	+	0/4	3/7
Progressive ophthalmoplegia	+	+	+	0/4	3/7
Seizures	−	−	−	1/4	1/7
Neonatal breathing abnormalities	−	−	−	0/4	0/7
**Skeletal Features**					
Mesoaxial polydactyly	+	−	−	4/4	5/7
Brachy- or clinodactyly V	+	−	+	1/4	3/7
Mild skeletal dysplasia	+	+	+	0/4	3/7
Narrow chest	−	−	−	1/4	1/7
Macrocephaly	−	−	+	2/4	3/7
Disproportionate short stature	−	−	−	0/4	0/7
**Orofacial Features**					
Oral clefts	−	−	−	1/4	1/7
Lustered hair, infantile onset	−	−	−	2/4	2/7
**Multisystemic Features**					
Cardiac defects	−	−	−	1/4	1/7
Renal involvement	−	−	−	0/4	0/7
Retinal involvement	−	−	−	0/1	0/4
Liver involvement	−	−	−	0/4	0/7
Sensorineuronal deafness	+	−	−	1/4	2/ 7
Lateralization defects	−	+	−	0/4	1/7
**Miscellaneous**					
	Myopia, cataract				

Abbreviations Table 1: DD, developmental delay; f, female; ID, intellectual disability; m, male; MTS, Molar tooth sign; OMA, Ocular motor apraxia; y, years; +, present; -, absent.

**Table 2:** Clinical features, diagnostic clues and pitfalls for *FAM149B1*, *POLG2* and *KMT2B* related multiple genetic disease in five siblings.

	P1	P2	P3	P4	P5
Ciliopathy *FAM149B1*	+	+	+	−	−
Clues	⮚ Characteristic ciliopathy features: MTS, OMA, ataxia, developmental delay, skeletal dysplasia ⮚ History of neonatal onset of neurological and ocular symptoms ⮚ ID with discrepancy between speech comprehension and verbal abilities ⮚ Consanguinity as a risk factor for autosomal recessive disorders				
Pitfalls	⮚ Ptosis as an overlapping symptom between *FAM149B1*-related JS and POLG2-related PO ⮚ PO phenocopying mitochondriopathy and masking characteristic ciliopathy features such as OMA, strabismus, nystagmus ⮚ Missing MTS on MRI ⮚ PO as novel symptom in JS; Absence of mesoaxial polydactyly as novel symptom in *FAM149B1*-related JS				
**Mitochondriopathy *POLG2***	**+**	**+**	**−**	**+**	**+**
Clues	⮚ Juvenile onset of neurological and ocular symptoms, no history of neonatal symptoms ⮚ Exercise intolerance and exercise-induced lactate elevation				
Pitfalls	⮚ Progression of the phenotype with age ⮚ Normal lactate levels at rest				
**Dystonia *KMT2B***	**−**	**−**	**−**	**−**	**+**
Clues	⮚ Severe childhood onset dystonia with basal ganglia involvement on brain MRI ⮚ ID without discrepancy between speech comprehension and verbal abilities for discrimination to *FAM149B1*-related ID				
Pitfalls	⮚ De novo mutation, thus molecular diagnosis has to be established in this patient				
**Sex**	M	F	M	F	M
**Current age**	40 y	25 y	18 y	26 y	21 y
**Ocular features**					
*Oculomotor disturbances*					
Strabism congenital onset	+	+	+	−	−
Intermittend strabism juvenile onset	−	−	−	+	+
Nystagmus, vanishing with age due to PO	+	+	+	−	−
Duane syndrome vanishing with age due to PO	+	+	+	−	−
PO	+ Complete age 35 y	+ incomplete age 24 y	+ incomplete age 18 y	−	−
Ptosis	+ 5 mm	−	−	−	−
*Ocular findings*					
Visual acuity (Snellen decimal) RE/LE	0.3/0.3	0.2/0.5	0.6/0.5	1.0/1.0	0.8/0.8
Refraction (diopters, spherical equivalent) RE/LE	−12.0/−12.0	+0.5/+0.5	−0.25/−0.25	+0.25/+0.25	−1.0/−1.0
Anterior segment	Mild lens opacification; BE	normal; BE	normal; BE	normal; BE	normal; BE
Fundoscopy	Myopic fundus degeneration with thinning of the retinal pigment epithelium and chorioid, peripapillariy myopic conus; BE	Mild situs inversus of the retinal vessels showing nasalward emergence from the optic disc; BE	normal; BE	normal; BE	normal; BE
Optical coherence tomography (macula)	Myopic thinning of outer retinal layers and choriocapillaris; BE	normal; BE	normal; BE	normal; BE	normal; BE
Full-field ERG (ISCEV Standard)	Dark and light adapted ERGs show subnormal amplitudes with normal timing in keeping with high myopia; BE	normal; BE	normal; BE	normal; BE	not feasible due to dystonia; BE
Pattern VEP	Not gradable due to loss of fixation/strabism; BE	normal; BE	normal; BE	normal; BE	not feasible due to dystonia; BE
Miscellaneous	Squint surgery; age 22 y				
**Neurological features**					
Movement disorder					
Cerebellar ataxia	+	+	+	−	−
Hypo-/Bradykinesia	+	+	+	−	−
Myoclonus	−	−	−	+	−
Tremor	−	−	−	−	Dystonic tremor
Athetosis/Dystonia	−	+	+	−	+++
Dysarthria	−	+	+	−	+
Myopathy or exercise intolerance and exercise induced lactat elevation	+	+	−	+	+
Infantile muscular hypotonia	+	+	+	−	−
Motor skill delay	+	+	+	−	−
Intellectual disability/IQ	+/ND	+/IQ 51	+/IQ 62	−/ND	+/IQ 64
Discrepancy between speech comprehension and verbal abilities	+	+	+	−	−
GDI score by gait analysisGDI normal range = 100	28	ND	74	ND	94
**Skeletal phenotype**					
Mild skeletal dysplasia with abnormal gait	+	+	+	−	−
Trident acetabular roofs with protrusion acetabuli	+	+	+	−	−
Premature and overt calcification of costal cartilage	+	+	−	−	−
Increased hip rotation	+	+	+	−	−
Increased external feet rotation	+	+	+	−	−
Dysproportionate stature	−	−	−	−	−
Macrocephaly	−	−	+	−	−
**Brain MRI**					
Molar tooth sign	+	+	+	−	−
Cerebellar hypoplasia of the upper vermis	+	+	+	−	−
Corpus callosal dysgenesis	+	−	+	−	−
Olfactory aplasia	+	+	−	−	−
Basal ganglia abnormalities	−	−	−	−	+
**Additional features**					
Endocrine abnormalities	−	−	−	−	−
Hearing loss	High frequency spectrum bilateral age 40 y	−	−	+ unilateral at age 26 y	+
Dysphagia adult onset	?	−	−	+	+
Sleep disturbance	+	−	−	+	−
Facial dysmorphism	−	−	−	−	−

Abbreviations Table 2: BE, both eyes; f, female; GDI, Gait Deviation Score; ID, intellectual disability; IQ, intelligence quotient; JS, Joubert syndrome; m, male; MRI, magnetic resonance imaging; MTS, Molar tooth sign; ND, not determined; OMA, ocular motor apraxia; PO, progressive ophthalmoplegia; RE/LE, right/left eye; y, years; +, present; +++, present, severe; −, absent.

## Data Availability

The data that support the findings of this study are available from the corresponding author, (J.V.), upon reasonable request.

## Supplementary Materials

The following are available online at https://www.mdpi.com/article/10.3390/genes12111648/s1, Table S1: Whole exome sequencing statistics in P3 and P5; Table S2: Whole exome sequencing variant filtering in P3 and P5.
