# Supplementary material for: Expanding the Phenotype of the FAM149B1-Related Ciliopathy and Identification of Three Neurogenetic Disorders in a Single Family
Source: Genes (Basel). 2021 Oct 20;12(11):1648. doi: 10.3390/genes12111648 (PMC8622907; doi:10.3390/genes12111648)
Supplement: Supplementary file 1 [file genes-12-01648-s001.zip › genes-1416671-SI.pdf]

SUPPLEMENTAL INFORMATION

Expanding the phenotype of the *FAM149B1*-related ciliopathy and identification of three neurogenetic disorders in a single family

Running title: *FAM149B1* ciliopathy in adults

Sandy Siegert,[1] Gabriel T. Mindler,[2,3] Christof Brücke,[4] Andreas Kranzl,[2,5] Janina Patsch,[3,6] Markus Ritter,[7] Andreas R. Janecke,[8,9] Julia Vodopiutz[1,3]

1 Department of Pediatrics and Adolescent Medicine, Division of Pediatric Pulmonology, Allergology and Endocrinology, Comprehensive Center for Pediatrics, Medical University of Vienna, 1090 Vienna, Austria

2 Department of Pediatric Orthopaedics, Orthopaedic Hospital Speising, 1130 Vienna, Austria

3 Vienna Bone and Growth Center

4 Department of Neurology, Medical University of Vienna, 1090 Vienna, Austria

5 Laboratory for Gait and Movement Analysis, Orthopaedic Hospital Speising, 1130 Vienna, Austria

6 Department of Biomedical Imaging and Image-Guided Therapy, Medical University of Vienna, 1090 Vienna, Austria

7 Department of Ophthalmology, Medical University of Vienna, Waehringer Guertel 18-20, 1090, Vienna, Austria

8 Department of Pediatrics I, Medical University of Innsbruck, 6020 Innsbruck, Austria

9 Division of Human Genetics, Medical University of Innsbruck, 6020 Innsbruck, Austria

SUPPLEMENTARY METHODS

Clinical examination

P1-P5 were evaluated at the Medical University of Vienna by a pediatrician-clinical geneticist (JV) and a neurologist (SS, CB). Examinations included medical and family history, physical examination, review of past medical records, brain MRI, skeletal radiographs and a blood and urine chemistry analysis. In P2, P3, and P5, assessment of intellectual functioning was performed, applying the Kaufman Assessment Battery for Children (K-ABC II, German version) in P3 at 17 years of age and the Wechsler Adult Intelligence Scale (WAIS, fourth revision, German version) in P2 and P5. All siblings and the mother underwent a physical examination, medical history and blood chemistry analysis.

Ocular examination

P1-5 underwent a complete ophthalmic examination, including best-corrected visual acuity, neuroorthoptic assessment, slit-lamp biomicroscopy and dilated fundus examination. Fundus photographs were obtained by ultra-wide field (up to 200°) confocal laser scanning ophthalmoscopy (Optos plc). Spectral domain optical coherence tomography (OCT) was performed in using a HRA+OCT Spectralis (Heidelberg Engineering, Heidelberg, Germany). Full-field electroretinography (ERG) and pattern visual evoked potential (PVEP) were performed to incorporate the International Society for Clinical Electrophysiology of Vision Standards. [1,2]

Computer based gait analysis

P1, P3, and P5 underwent a gait analysis in the Laboratory for Gait and Movement Analysis (Orthopaedic Hospital Speising, Vienna) using a modified Cleveland model combined with a Plug in Gait model.[3,4] A motion capture system (Vicon, Oxford, United Kingdom) with 17

cameras and force plates were used. Patients walked a 12-meter walkway and kinematic and kinetic parameters of thorax, pelvis, hip, knee and ankle were measured. The GDI (Gait Deviation Score) was calculated according to Schwartz and Rozumalski.[5]

SUPPLEMENTARY RESULTS

The vcf files generated by the WES variant calling pipeline in P3 and P5 were submitted to SeattleSeq (http://snp.gs.washington.edu/SeattleSeqAnnotation/) for annotation, categorization, and filtering against public variant databases, and the following output was obtained: Supplementary Table 1, 2

SUPPLEMENTARY TABLES

Supplementary Table S1. Whole exome sequencing statistics in P3 and P5

Sample*	P3	P5
**Total reads**	78021084	68130514
**% mapped reads**	94.17	95.52
**Mapped bases on target**	3953410068	4840223077
**Mean coverage (X)**	90.52	83.7
**% target bases covered = 0X**	1.47	2.0
**% target bases covered ≥ 2X**	98.2	97.57
**% target bases covered ≥ 10X**	96.33	96.42
**% target bases covered ≥ 20X**	93.74	94.45
**% target bases covered ≥ 50X**	77.6	78.42
**% target bases covered ≥ 100X**	36.0	29.41

Supplementary Table S2. Whole exome sequencing variant filtering in P3 and P5

P3	P5
# Count missense SNVs = 10488	# Count missense SNVs = 9409
# Count stop SNVs = 116	# Count stop SNVs = 92
# Count SNVs in splice sites = 82	# Count SNVs in splice sites = 52
# Count SNVs in coding synonymous = 10971	# Count SNVs in coding synonymous = 10171
# Count SNVs in coding (not mod 3) = 58	# Count SNVs in coding (not mod 3) = 54
# Count SNVs in a UTR = 7392	# Count SNVs in a UTR = 2516
# Count SNVs near a gene = 13310	# Count SNVs near a gene = 879
# Count SNVs in introns = 139440	# Count SNVs in introns = 22984
# Count intergenic SNVs = 100473	# Count intergenic SNVs = 1696
# Count frameshift indels = 63	# Count frameshift indels = 47
# Count indels coding-not-frameshift or coding-unknown = 590	# Count indels coding-not-frameshift or coding-unknown = 455
# Count indels in splice sites = 19	# Count indels in splice sites = 7
# Count indels in a UTR = 1149	# Count indels in a UTR = 299
# Count indels near a gene = 2804	# Count indels near a gene = 77
# Count indels in introns = 24678	# Count indels in introns = 2692
# Count intergenic indels = 19957	# Count intergenic indels = 131
# number SNPs in dbSNP = 299912	# number variants in dbSNP = 51441
# number SNPs not in dbSNP = 35095	# number variants not in dbSNP = 940
# number SNPs total = 335007	# number variants total = 52381

SUPPLEMENTARY REFERENCES

1. Odom JV, Bach M, Brigell M, et al. ISCEV standard for clinical visual evoked potentials: (2016 update). *Doc Ophthalmol.* 2016;133(1):1-9.
2. McCulloch DL, Marmor MF, Brigell MG, et al. ISCEV Standard for full-field clinical electroretinography (2015 update). *Doc Ophthalmol.* 2015;130(1):1-12.
3. A.Kranzl BS. A study of the reproducibility of the marker application of the Cleveland Clinic Marker Set including the Plug-In Gait Upper Body Model in clinical gait analysis. *Gait & Posture.* 2012;Volume 36(June 2012):Pages S62-S63.
4. Sutherland DH. The evolution of clinical gait analysis. Part II kinematics. *Gait Posture.* 2002;16(2):159-179.
5. Schwartz MH, Rozumalski A. The Gait Deviation Index: a new comprehensive index of gait pathology. *Gait Posture.* 2008;28(3):351-357.
